# Autonomic Dysfunction in α-Synucleinopathies

**DOI:** 10.3389/fneur.2019.00363

**Published:** 2019-04-12

**Authors:** José Javier Mendoza-Velásquez, Juan Francisco Flores-Vázquez, Evalinda Barrón-Velázquez, Ana Luisa Sosa-Ortiz, Ben-Min Woo Illigens, Timo Siepmann

**Affiliations:** ^1^Division of Health Care Sciences, Center for Clinical Research and Management Education, Dresden International University, Dresden, Germany; ^2^Department of Psychiatry and Mental Health, School of Medicine, National Autonomous University of Mexico, Mexico City, Mexico; ^3^Dementia Laboratory, National Institute of Neurology and Neurosurgery, Ciudad de Mexico, Mexico; ^4^Faculty of Medical Sciences, University of Groningen, Groningen, Netherlands; ^5^Department of Neurology, Beth Israel Deaconess Medical Center, Harvard Medical School, Boston, MA, United States; ^6^Department of Neurology, University Hospital Carl Gustav Carus, Technische Universität Dresden, Dresden, Germany

**Keywords:** autonomic dysfunction, α-synucleinopathies, Parkinson disease, dementia with Lewy bodies, multiple system atrophy, pure autonomic failure, dysautonomia

## Abstract

The α-synucleinopathies are a group of neurodegenerative diseases characterized by abnormal accumulation of insoluble α-synuclein in neurons and glial cells, comprising Parkinson's disease (PD), dementia with Lewy bodies (DLB) and multiple system atrophy (MSA). Although varying in prevalence, symptom patterns, and severity among disorders, all α-synucleinopathies have in common autonomic nervous system dysfunctions, which reduce quality of life. Frequent symptoms among α-synucleinopathies include constipation, urinary and sexual dysfunction, and cardiovascular autonomic symptoms such as orthostatic hypotension, supine hypertension, and reduced heart rate variability. Symptoms due to autonomic dysfunction can appear before motor symptom onset, particularly in MSA and PD, hence, detection and quantitative analysis of these symptoms can enable early diagnosis and initiation of treatment, as well as identification of at-risk populations. While patients with PD, DLB, and MSA show both central and peripheral nervous system involvement of α-synuclein pathology, pure autonomic failure (PAF) is a condition characterized by generalized dysregulation of the autonomic nervous system with neuronal cytoplasmic α-synuclein inclusions in the peripheral autonomic small nerve fibers. Patients with PAF often present with orthostatic hypotension, reduced heart rate variability, anhydrosis, erectile dysfunction, and constipation, without motor or cognitive impairment. These patients also have an increased risk of developing an α-synucleinopathy with central involvement, such as PD, DLB, or MSA in later life, possibly indicating a pathophysiological disease continuum. Pathophysiological aspects, as well as developments in diagnosing and treating dysautonomic symptoms in patients with α-synucleinopathies are discussed in this review.

## Introduction

The α-synucleinopathies are neurodegenerative diseases charac-terized by the abnormal accumulation of α-synuclein aggregates in neurons and glial cells. These include, in order of prevalence: Parkinson's disease (PD), dementia with Lewy bodies (DLB), and multiple system atrophy (MSA), as well as various rare neuroaxonal dystrophies (1). A highly related condition, pure autonomic failure (PAF), features generalized dysregulation of the autonomic nervous system, with neuronal cytoplasmic α-synuclein inclusions in the peripheral autonomic small nerve fibers, and is regarded as a high-risk condition to develop PD, DLB, or MSA (2, 3).

Dysautonomic symptoms are frequently found in the various α-synucleinopathies, and can occur in any stage of the disease, even in their prodromal states. Autonomic dysfunction includes symptoms such as orthostatic hypotension (OH), reduced heart rate variability, supine hypertension, constipation, fecal incontinence, urinary, and sexual dysfunction. These symptoms are originated by the damage to distinct components of the central and peripheral autonomic nervous system (4–7).

Deposits of α-synuclein accumulate first in peripheral nerves, including those in the skin and enteric mucosa, advancing toward the brain through the vagal and olfactory nerves and progressing through the encephalon, in a determined pattern according to the particular disease phenotype (1, 8–10). This pathological progression can explain the early apparition of non-motor symptoms, among them, autonomic nervous system dysfunction (11, 12).

Dysautonomic manifestations of the specific α-synucleinopathies are caused by the involvement of various components of the autonomous nervous system. In PD, cardiovascular autonomic dysfunction is related to a loss of peripheral noradrenergic innervation, while constipation most likely reflects direct involvement of the enteric nervous system neurons. In MSA, dysautonomic symptoms are mostly related to degeneration of preganglionic autonomic neurons of the brainstem and spinal cord (13).

Recently, the identification of α-synuclein deposits in skin biopsies has opened a window to better understand autonomic denervation, as well as providing a sensitive and specific biomarker for early diagnosis of the α-synucleinopathies, with a strong correlation between α-synuclein load in cutaneous small fibers and measures of cardiovascular autonomic function, and skin pilomotor and sudomotor responses (10, 14–17).

## Prevalence and Impact of Autonomic Dysfunction in α-Synucleinopathies

PD is the second most-common neurodegenerative disease, affecting 2–3% of the population above 65 years of age (18). The prevalence of autonomic dysfunction in PD ranges between 50 and 70% (19–21). The most common dysautonomic symptoms in PD are constipation, urinary dysfunction, and OH (20). Dysautonomic symptoms have been proposed as part of the criteria for prodromal PD, together with REM sleep behavior disorder, molecular neuroimaging biomarkers, sub-threshold parkinsonism, hyposmia, depression, and anxiety (12, 22). In PD, dysautonomic syndromes have a heterogeneous presentation, and their progression is not predictable, however, their presence is associated with a deterioration in autonomy and quality of life, regardless of the duration of the disease, cognitive decline, or the severity of motor symptoms (5, 23).

DLB is the second most frequent neurodegenerative dementia, affecting up to 0.7% of the population above 60 years of age, and causing up to 24% of the total cases of dementia worldwide (24). Dysautonomic symptoms are a part of the supportive clinical features for the diagnostic criteria of this disease, and their estimated prevalence is 62% (21, 25, 26). In DLB, autonomic dysfunction can be a prodromal feature (11): in a case series of 90 patients with DLB, more than half displayed dysautonomic symptoms (particularly OH) prior to the onset of cognitive impairment (27).

MSA is an infrequent cause of dementia, with an incidence of 3 per 100,000 person-years in people above 50 years of age (28). Dysautonomia is a core clinical criteria for this condition, which subdivides into two phenotypes, depending on the predominance of motor symptoms (cerebellar or parkinsonian), additional to autonomic dysfunction (29). Autonomic dysfunction can precede the onset of motor symptoms of MSA in up to 50% of patients (30). Urinary dysfunction and OH are the most frequent dysautonomic symptoms of MSA, with an earlier onset of urinary symptoms, particularly in the cerebellar phenotype (30, 31). In MSA, severe dysautonomia and the early combination of dysautonomic and motor symptoms are poor prognostic factors, regardless of the phenotype (32).

A syndrome that deserves special attention in the study of α-synucleinopathies is pure autonomic failure (PAF). PAF is defined by the presence of chronic OH, without clinical signs of central neurodegeneration (2, 33). Patients with PAF can also display supine hypertension, constipation, urinary symptoms and thermic dysregulation (7). In a 4-year follow-up study of 100 patients with PAF, 34% progressed to an α-synucleinopathies. The risk of conversion was seven times higher in subjects that, in addition to dysautonomic symptoms, presented a REM sleep behavior disorder. Patients that progressed to PD or DLB had a higher prevalence of hyposmia, worse response to the head-up tilt test, and a longer disease course; while those that converted to MSA had a younger onset dysautonomia and a higher prevalence of urinary and bowel dysfunction. The subjects that did not convert to any of these diseases had significantly lower levels of blood epinephrine (6). α-synuclein has also been found in skin biopsies and postganglionic sympathetic neurons of PAF patients, reflecting a common pathological precursor between PAF and other α-synucleinopathies (13, 34). An autonomic-only presentation of MSA can be indistinguishable from PAF, specially in the early stages (35).

## Specific Dysautonomic Symptoms in α-Synucleinopathies

OH is the main clinical feature of cardiovascular autonomic dysfunction, and it is defined as sustained drop in systolic pressure of at least 20 mm Hg and/or a sustained diastolic drop of at least 10 mm Hg within the first 3 min after standing up (36, 37). This time cut-off might not be sensitive for α-synucleinopathies, in which the presentation is most commonly that of delayed OH, therefore, measuring blood pressure for at least 10 min has been recommended (36). Delayed OH has been documented as a risk factor for α-synucleinopathies, and frequently progresses to OH with a high associated mortality (38). Noradrenergic cardiac and extracardiac denervation, as well as the lack of arterial baroreflexes in α-synucleinopathies are causal factors of this symptom (39, 40). The loss of baroreceptor sensitivity has been documented through spectral analysis of heart rate (R-R interval) and systolic arterial pressure variability in PAF (41) and PD (42), even before the onset of OH (43, 44). A functional association between OH and cognitive decline in α-synucleinopathies has been documented, given that OH aggravates neural damage because of cerebral hypoperfusion (36, 45, 46). OH affects 30–60% of PD patients, and has been linked to an elevated frequency of falls, detriment of physical activity, and use of health care services, even if OH is asymptomatic (23, 29, 47, 48). The frequency of OH varies according to the stage of the disease, from 14% in early-stage PD patients to 52% in later cases or older individuals (49–51). Around 68% of patients with DLB display OH, and about 17% suffer associated syncope (26, 36, 52). OH affects around 43% of patients with MSA from early stages of the disease, and of these, 50% also display post-prandial hypotension, as well as nocturnal and supine hypertension (30, 53, 54). This condition is more frequent and more severe in the cerebellar phenotype of MSA when compared to the parkinsonian subtype (55).

Constipation is defined as a frequency of less than three bowel movements in 1 week (56). Between 54 and 90% of PD patients suffer from constipation, and out of these, 48% report the onset of constipation up to 10 years prior to the onset of motor symptoms (20, 57, 58). Constipation is more frequent in patients with a rigid-bradykinetic phenotype and is related to the presence of neuropsychiatric symptoms, such as anxiety, depression, and insomnia (57). Constipation may start even before in MSA than in PD (59). In patients with DLB, a frequency of 30% has been documented (60).

In patients with α-synucleinopathies, the gastrointestinal function is disturbed at all levels. Dysphagia in PD and DLB tends to be mild, and appears in later stages of the disease, while in MSA it can be early and severe. Aspiration pneumonia is a common cause of death in α-synucleinopathies, and higher gastrointestinal symptoms (attributable to esophageal dysmotility and gastroparesis) diminish the quality of life of these patients (58, 61).

Urinary dysfunction is present in up to 71% of PD patients, mostly with nocturia and altered urinary frequency (62). In a Japanese study that included 32 patients with DLB, a 90% prevalence of urinary dysfunction was found, with a predominance of nocturia, followed by urinary incontinence and detrusor hyperactivity (63). Up to 96% of MSA patients display urinary symptoms, which tend to be more severe than in PD, and 60% start before the onset of motor symptoms (with a mean of 4 years before diagnosis), mostly with post-residual volume alterations (53, 62, 64).

Erectile dysfunction is defined as the incapacity to achieve or maintain a penile erection long enough to allow a sexual relation (65). In a 7-year follow-up study of 3,153 patients with erectile dysfunction, a 1.52-times higher risk of PD was found, with an even higher risk if cardiovascular risk factors, such as diabetes or hypertension, was concurrent (66). Erectile dysfunction is present in up to 97% of men diagnosed with MSA, and it is the initial symptom in 48% of male patients, preceding motor symptoms for as long as a decade (30, 59, 64). Female sexual dysfunction has been less studied in α-synucleinopathies, but a higher prevalence of this disorder has been found in female PD patients than in age-matched controls, and it is related to older age and a higher severity of depressive symptoms (67).

## Clinical Assessment of Autonomic Dysfunction in α-Synucleinopathies

Clinical tests designed to measure the end-organ responses to the autonomic nervous systems can be used to quantitatively analyze autonomic dysfunction, playing an important role in the clinical assessment of α-synucleinopathies.

Tests of cardiovagal function include heart rate variability with deep breathing, postural changes (such as the head-up tilt test), or the Valsalva maneuver, in which the patient forcefully exhales into a sphygmomanometer with an open glottis at a pressure of 40 mmHg for 15 s. Sympathetic adrenergic function can be assessed by measuring blood pressure response to postural change, Valsalva maneuver or isometric exercise, as well as by the cold pressor test, in which the subject is instructed to immerse his or her hand in ice water for 1 min (68). The decrease of heart rate and blood pressure variability can be accurately demonstrated through power spectrum techniques, which provide a quantitative assessment of said variability (41, 43, 69, 70). Ambulatory blood pressure monitoring can also provide sensitive markers of autonomous nervous system failure, such as post-prandial hypotension and nocturnal/supine hypertension (71, 72).

Clinical assessments of sudomotor function include thermoregulatory sweat testing, quantitative sudomotor axon reflex testing, silicone impression, the sympathetic skin response, the acetylcholine sweat-spot test, and quantitative direct and indirect axon reflex testing, as well as electromyographic skin potentials (73, 74). Cutaneous autonomic pilomotor testing, in which iontophoresis of phenylephrine induces a local neurogenic pilomotor erection (“goose bumps”) as a measure of functional integrity of autonomic skin nerve fibers, is an approach to capture the progression of autonomic nerve dysfunction and α-synuclein deposition (75).

Differential diagnosis of the parkinsonian subtype of MSA and PD or other parkinsonian syndromes is mostly based on the evaluation of autonomic dysfunction (9, 30). Clinical autonomic cardiovascular tests can distinguish MSA and PD with a sensitivity of 91% and a specificity of 92%. (123)-I-myocardial metaiodobenzyguanidine (MIBG) scintigraphy can distinguish these entities with a sensitivity of 90% and specificity of 82% (7, 30). Cardiovagal baroreflex is also sensitive for the differentiation between MSA and PD, being disproportionally affected in MSA (76). Added sweating and thermoregulation tests have also been found to improve differential diagnostic reliability (77, 78).

## Molecular and Cellular Aspects of Autonomic Dysfunction in α-Synucleinopathies

Mutations in the gene encoding for α-synuclein, SNCA, as well as in some of the genes collectively referred to as PARK (including the LRRK2 and VPS35 genes), have been associated with variants of autosomal dominant PD, and others such as PARK2, PINK1, and PARK7 to autosomal recessive PD. Although the mechanism has not been completely clarified, it is known that mutated proteins have different roles in autophagy and the degradation of nerve cells. Familial cases of DLB have been associated with mutations in the PARK, SNCA, SNCB, and LRRK2 genes. In the rare familial cases of MSA, there is a reported association to SNCA and COQ2 genes. Autonomic dysfunction has been associated with six SNCA mutations in different groups, including subjects with PAF prior to the onset of motor symptoms (79, 80).

The families carrying PD with a chromosomal triplication of SNCA present OH with evidence of sympathetic cardiac denervation and frequent associated falls up to 3 years before the onset of the disease. However, there are triplications of SNCA without documented autonomic dysfunctions. This phenotypic heterogeneity could be explained by the variability in the genomic size of SNCA triplications, meaning that different genes could be involved. In addition to OH, other dysautonomic symptoms, such as urinary incontinence and severe constipation of early onset, are more frequent in triplications of SNCA compared to duplications. A sympathetic cardiac denervation has also been found in heterozygous carriers with biallelic mutations of PARK2, causal of the most common autosomal recessive form of PD. Other mutations in the LRRK gene are also associated with different autonomic profiles in PD, with symptoms such as constipation, neurogenic bladder, and erectile dysfunction (79).

The central autonomic network and preganglionic sympathetic and parasympathetic neurons are variably affected in the different α-synucleinopathies. In PD and DLB, the dorsal motor nucleus of the vagus nerve, and in MSA the ventrolateral medulla, hypothalamus and preganglionic neurons are key structures affected that explain the origin of autonomic dysfunction (13). PAF involves generalized loss of sympathoadrenomedullary cells, as reflected by plasma levels of catechols and metanephrines, in contrast to MSA and PD, where adrenomedullary cells seem to remain intact, but organ-selective sympathetic denervation occurs (81). Furthermore, α-synuclein-containing glial cytoplasmic inclusions have been found in the Oluf's nucleus of MSA-affected individuals, which might account for early urinary, defecatory and sexual symptoms (64).

In brain tissue, an abnormal accumulation of α-synuclein has been found in the left posterior part of the insula of patients with PD, correlated with the presence of OH. The inclusions of α-synuclein in the hypothalamus of patients with PD may be linked to hypothalamic dysfunction, resulting from lesions in the thermoregulatory centers of the preoptic area, causing hypothermia, episodes of sweating, or hypohidrosis. In MSA, the neurons of the paraventricular nucleus project to the intermediolateral cell column, and their dysfunction can contribute to the lack of control of sympathetic function, causing OH. Both MSA and DLB show a loss of tyrosine hydroxylase in neurons of the periaqueductal gray matter, which is related to autonomic cardiovascular and urinary dysfunction (13). With respect to constipation and gastrointestinal symptoms presented by patients, α-synuclein inclusions have been detected in colon biopsies up to 8 years before the onset of motor symptoms of PD and, although the studies are not consistent, it is believed that the presence of extracellular α-synuclein is associated with acute and chronic inflammatory conditions of the intestine (82).

## Treatment of Autonomic Dysfunction in α-Synucleinopathies

Dysautonomic symptoms are among the most debilitating in α-synucleinopathies, but, when recognized, they can be treated using both pharmacological and non-pharmacological strategies, including the suspension of potentially causing or aggravating medications, and patient education. Table 1 shows the therapeutic strategies for this group of symptoms.

**Table 1:** Pharmacological and non-pharmacological strategies for dysautonomic symptoms in α-synucleinopathies.

**Dysautonomic symptom**	**Pharmacological strategy**	**Non-pharmacological strategy**
Orthostatic hypotension	Expansion of intravascular volume with fludrocortisone (58) Increase of peripheral vascular resistance with midodrine, droxidopa or norepinephrine transporter inhibitors, such as atomoxetine, yohimbine, ergotamine, and caffeine (58, 83) Potentiation of peripheral cholinergic neurotransmission (84) Domperidone in non-cardiac patients (85)	Discontinue antihypertensive and other medications that can cause orthostatic hypotension (84) Physical contermaneuvers (e.g., standing with legs crossed, squatting, active tensing of leg muscles, breathing-related maneuvers to increase inspiratory resistance, and avoiding getting up too quickly or standing motionless) (58, 86) Use of compression stockings (58) Increase the consumption of water and drinks with caffeine during meals (58, 86) Eat small, frequent meals (86) Physical activity such as water exercise, recumbent bicycling, or rowing (86) Avoid alcohol consumption (86) Avoid situations that increase core body temperature such as prolonged hot showers (86) Plantar mechanical stimulation is a promising approach for the regulation of heart rate variability in PD (42, 69)
Supine hypertension	Antihypertensives: captoptil, nevibolol, clonidine, hydralazine, losartan (58) Clonidine, nitroglycerin patches, and short-acting nifedipine (83, 84)	At night, tilt the bed to achieve an angle of 30 or 45 degrees (58) The application of abdominal local heat could be of benefit (58)
Constipation	Bulk laxatives, like psyllium or methylcellulose (58) Osmotic laxatives (polyethylene glycol, magnesium, lactuslose) (58)	Probiotics, high fiber diets, olive oil Adequate hydration (58) Physical activity (87)
Dysphagia and excessive salivation	Botulinum toxin in the distal esophagus could improve dysphagia (58) Vocal fold augmentation, including injection laryngoplasty (88) In patients with sialorrhea, treatment with glycopyrrolate and the local application of anticholinergics, as drops of sublingual atropine or ipatropium spray (58)	Reduce the volume of food (58) Eat slowly (58) Eat foods with a more liquid consistency (58) Speech and swallowing therapy (61, 89)
Gastroparesis	Dopamine blockers like metoclopramide, itopride (58) Motilin receptor agonists such as erythromycin (58) Serotonergic agonists like cisapride (58)	Low fat diet (58) Small but frequent meals (58)
Urinary dysfunction	B3-adrenergic agonists like mirabregon (58) Antimuscarinic agents such as oxybutynin, atripine, scopolamine (58) Alpha-adrenergic blockers like tamsolusin (58)	Biofeedback (58) Deep brain stimulation of the subthalamic nulcei (90)
Erectile dysfunction	Phosphodiesterase type 5 (PDE-5) inhibitors, with caution because of potentially severe hypotension (58) Intraurethral prostaglandin suppositories (58)	Psychotherapy, sex counseling seeking “pleasure oriented” activity instead of “goal-oriented” intercourse (91) Vacuum pump devices (58) Surgical placement of penis prosthesis (58)
Female sexual dysfunction	Hormonal replacement therapy (58)	Psychotherapy, sex counseling seeking “pleasure oriented” activity instead of “goal-oriented” intercourse (58, 91) Vaginal lubrication (58)

## Conclusions

Dysautonomic symptoms frequently occuring in α-synucleinopathies comprise cardiovascular, gastrointestinal, urogenital and thermoregulatory disturbances. These symptoms reduce quality of life and worsen prognosis. The understanding of their pathophysiology, as well as the detection of α-synuclein deposition and autonomic dysfunction in the premotor stages of α-synucleinopathies may be key for identifying novel treatment targets and improving clinical outcomes. While causative treatment is not yet available, improvement of quality of life can be achieved by personalized symptomatic treatment regimens, which includes both and pharmacological and non-pharmacological strategies.

## Author Contributions

JM-V: oversight of teamwork, literature search and review of articles, writing of abstract and parts 2, 3, and 4; JF-V: literature search and review of articles, writing of parts 1, 2, and 4; EB-V: review of articles, writing of parts 5 and 6; AS-O and B-MI: proofreading, expert advice on theoretical and clinical aspects; TS: proofreading, expert advice on theoretical and clinical aspects, identification of additional relevant papers.

### Conflict of Interest Statement

The authors declare that the research was conducted in the absence of any commercial or financial relationships that could be construed as a potential conflict of interest.
